# Medical Preceptors and College Professors

**Published:** 1881-01

**Authors:** William R. Munroe

**Affiliations:** Baltimore, Md.


ARTICLE V.

### MEDICAL PRECEPTORS AND COLLEGE PROFESSORS.

BY WILLIAM R. MUNROE, M. D., BALTIMORE, MD.

It has often been said, and with much truth, that second only to the ministry is the medical profession, in its sacredness among families and in home circles. Because of his opportunities, the physician's responsibility is almost above and beyond that of the minister, both in a physical and moral sense, for prudential reasons, in extreme illness. For he is the only one who can administer consolation to his suffering patient, as he only has access to the couch of the ill person in the family. A human life, yea, a human soul, with its fearful destinies, is in his hands, for all others it may be admission is interdicted. Ought not the family physician, in view of these responsibilities, to be a good man, a true man and a righteous man?

As a general rule, *as is the teacher so is the pupil;* or, as is the preceptor so is the student! The morals of medical students will hardly rise higher than those of their professors at college. In large cities like Baltimore, where hundreds of young men attend the medical lectures every winter, many of whom are from the country, or small towns, and are the sons of pious parents, and they necessarily become exposed to many temptations unknown to them at their homes. When a medical faculty, composed in part, or in whole, of *skeptical* and *immoral* professors, who fear not God, nor regard the good wishes of Christian parents for the moral welfare of their sons, but introduce into their lectures, ever and anon, obscene anecdotes, and unchaste language, couched in unnecessary vulgar utterances, it tends to encourage immorality in the students, and to lower the dignity as well as to debase the sacredness of the medical profession!

*All* of our medical preceptors and college professors, should be gentlemen, high-toned, and dignified *Christian men*, as well as skilled medical teachers.

Is it not high time that *Christian parents* who wish to give their sons a *good medical education* should *inquire* into the *characters of all the men composing the faculty* of a medical school, before deciding to send a son or ward to it for his education, even though the fees should be of merely nominal amount?

What parent, wishing to prepare his son for the Christian ministry, or for some other profession than that of medicine, will not be very careful that the college into which he enters shall have a faculty composed of men thoroughly orthodox and of undoubted high moral characters? Why, then, should students of medicine be permitted recklessly to enter a medical school whose professors are of unknown, or doubtful moral and Christian qualities?

